# The role of ultrasonographic lung aeration score in the prediction of postoperative pulmonary complications: an observational study

**DOI:** 10.1186/s12871-021-01236-6

**Published:** 2021-01-14

**Authors:** Marcell Szabó, Anna Bozó, Katalin Darvas, Sándor Soós, Márta Őzse, Zsolt D. Iványi

**Affiliations:** 1 1st Department of Surgery and Interventional Gastroenterology, Semmelweis University, 1082 Üllői út 78, Budapest, Hungary; 2 Department of Anesthesiology and Intensive Therapy, Semmelweis University, 1082 Üllői út 78B, Budapest, Hungary

**Keywords:** Lung ultrasound, Point-of-care ultrasound, Postoperative pulmonary complications, Ultrasonography, Perioperative care

## Abstract

**Background:**

Postoperative pulmonary complications (PPCs) are important contributors to mortality and morbidity after surgery. The available predicting models are useful in preoperative risk assessment, but there is a need for validated tools for the early postoperative period as well. Lung ultrasound is becoming popular in intensive and perioperative care and there is a growing interest to evaluate its role in the detection of postoperative pulmonary pathologies.

**Objectives:**

We aimed to identify characteristics with the potential of recognizing patients at risk by comparing the lung ultrasound scores (LUS) of patients with/without PPC in a 24-h postoperative timeframe.

**Methods:**

Observational study at a university clinic. We recruited ASA 2–3 patients undergoing elective major abdominal surgery under general anaesthesia. LUS was assessed preoperatively, and also 1 and 24 h after surgery. Baseline and operative characteristics were also collected. A one-week follow up identified PPC+ and PPC- patients. Significantly differing LUS values underwent ROC analysis. A multi-variate logistic regression analysis with forward stepwise model building was performed to find independent predictors of PPCs.

**Results:**

Out of the 77 recruited patients, 67 were included in the study. We evaluated 18 patients in the PPC+ and 49 in the PPC- group. Mean ages were 68.4 ± 10.2 and 66.4 ± 9.6 years, respectively (*p* = 0.4829). Patients conforming to ASA 3 class were significantly more represented in the PPC+ group (66.7 and 26.5%; *p* = 0.0026). LUS at baseline and in the postoperative hour were similar in both populations. The median LUS at 0 h was 1.5 (IQR 1–2) and 1 (IQR 0–2; *p* = 0.4625) in the PPC+ and PPC- groups, respectively. In the first postoperative hour, both groups had a marked increase, resulting in scores of 6.5 (IQR 3–9) and 5 (IQR 3–7; *p* = 0.1925). However, in the 24th hour, median LUS were significantly higher in the PPC+ group (6; IQR 6–10 vs 3; IQR 2–4; *p* < 0.0001) and it was an independent risk factor (OR = 2.6448 CI95% 1.5555–4.4971; *p* = 0.0003). ROC analysis identified the optimal cut-off at 5 points with high sensitivity (0.9444) and good specificity (0.7755).

**Conclusion:**

Postoperative LUS at 24 h can identify patients at risk of or in an early phase of PPCs.

**Supplementary Information:**

The online version contains supplementary material available at 10.1186/s12871-021-01236-6.

## Background

Postoperative pulmonary complications (PPCs) are important causes of mortality after major noncardiac surgeries, and they adversely affect several aspects of morbidity, including the length of hospital stay and unexpected intensive care unit admissions [1–4]. Their incidence is reported in a wide range (2.8–40%) depending mostly on the represented patient population and PPC definitions [1, 2, 4–8]. Even though no single universal definition exists, there is a widespread consensus about the involvement of the following in its description: respiratory infection, respiratory failure, bronchospasm, atelectasis, pleural effusion, pneumothorax, or aspiration pneumonitis [1, 2, 6, 7] while others add pulmonary oedema and tracheal reintubation as well [9]. Several risk stratification models have been described [2, 4, 8, 10], identifying patient- and procedure-related predictors. Risk stratification can identify high-risk patients, but there is a lack of validated tools in monitoring patients for early stages of developing PPCs in a potentially reversible phase. However, conventional chest X-rays remained routine in thoracic diagnostics, but the widespread use of ultrasound by anaesthesiologists and intensive care physicians made this modality a real point of care alternative. As a non-invasive tool offering practically unlimited repetitions, ultrasound became a valuable method in critical care for the assessment of pleural effusions [11], pneumothorax [12, 13], and complex protocols exist to diagnose the various causes of respiratory insufficiency or cardiac arrest [14]. The perioperative use also seems sensitive and specific for PPCs. Lung ultrasound is reported to be superior to radiography in detecting any of the PPCs after cardiothoracic surgery [15]. A quantitative scoring system originally described by Bouhemad et al. was effectively used to drive ventilation strategy in ARDS patients or to predict weaning failure [16–20]. This scoring system relies heavily on ‘B-lines’; their increased numbers and subsequently confluent profiles are threshold steps in forming categories. B-lines are discrete laser-like vertical hyperechoic reverberation artifacts arising from the pleural line (previously described as ‘comet tails’), extend to the bottom of the screen without fading, and move synchronously with lung sliding [21]. They are considered to be corresponding to widened interlobular septa and can appear bilaterally, conforming to the diagnosis of interstitial syndrome of the lung including pulmonary oedema irrespective to its cause [12, 22], but non-symmetric appearance can be linked to other causes of decreased lung aeration or to interstitial pulmonary diseases [19, 23]. Although, by nature, this system uses few categories, good correlation was verified compared to data from hemodynamic monitoring by pulmonary catheterisation (wedge pressure) or thermodilution (extravascular lung water) [24], and verification studies by CT are also available [16, 25]. Monastesse et al. verified that, with minor modifications, it is also feasible for perioperative lung aeration assessment [26].

The aim of the present study was to evaluate the role of the lung aeration score measured on definite timepoints of the first 24 h after major abdominal surgery in the prediction of developing PPCs.

## Materials and methods

### Patients

This prospective, observational study was conducted between 25/08/2019 and 24/07/2020 in the 1st Department of Surgery, Semmelweis University, Budapest, Hungary. Ethics approval for this study was provided by Semmelweis University Regional and Institutional Committee of Science and Research Ethics, Budapest, Hungary (Registration number: SE RKEB 158/2019, date of approval: 31/07/2019). Informed consent was obtained from each subject. Subjects were ≥ 18 years, ASA 2 or 3 classified patients, who were scheduled for elective major abdominal surgery under general anaesthesia with endotracheal intubation on predetermined weekdays. Major surgery was defined as predicted duration of ≥120 min, expected need for postoperative intensive therapy or high dependency care, operations involving the thoracic cavity were excluded. The prediction of the operation time and booking for ICU/HDU beds depended on the judgement of the attending surgeons and anaesthesiologists. Inclusion and exclusion criteria are detailed in Table 1.

**Table 1:** Inclusion and exclusion criteria of the study

Inclusion criteria	Exclusion criteria
Age ≥ 18 years ASA class 2 or 3 Major abdominal surgery General anaesthesia	Preceding surgery within 30 days Thoracotomy History of lung resection Oxygen therapy at rest Any kind of acute pulmonary morbidity Patient on ventilatory support at surgical admission

*ASA* American Society of Anaesthesiology

Baseline characteristics such as comorbidity data, basic demographic data, and ASA class were recorded. Comorbidity data included history of hypertension, chronic obstructive pulmonary disease (COPD), congestive heart failure (irrespective of EF), diabetes (any type), smoking status by self-report, and active extrapulmonary infection. Preoperative oxygen saturation was recorded on the day of surgery on ambient air. Commonly available biomarker levels with literature relevance as predictors for PPC, such as haemoglobin level and creatinine were also collected [2, 4].

The data on the surgical procedure included the type of surgery, duration of the procedure, and epidural use. We also assumed operative fluid balance [27], which was calculated from intravenous fluid therapy, urine output and content of the suction vessel with surgical sponges (where used) without the quantity of saline used for lavage.

ARISCAT (Assess Respiratory Risk in Surgical Patients in Catalonia) score, a cumulative determinant of PPC risk was also calculated [2].

### Ultrasound protocol

Ultrasonographic scans were performed by one of four adequately trained independent anaesthesiologists who had undergone institutional training for ultrasound use in anaesthesia, and who had at least 2 years of experience in the field conforming to the criteria of adequate experience validated in LUS training programs [28]. All examinations were performed using the same ultrasound machine (Hitachi Aloka Noblus, Hitachi Healthcare, Tokyo, Japan). A linear transducer of 10–3 MHz was selected, a study preset of 7.5 MHz without tissue harmonic imaging was activated, and care was taken on focus positioning to the proximity of the parietal pleura. In particular cases, the ultrasonographer could choose a convex probe of 5 MHz to obtain images from obese patients [29].

Patients were examined in semirecumbent position. Six fields of each hemithorax were scanned defined by the mamillar line horizontally, the anterior and posterior axillary lines vertically, following a similar approach used in previous studies [16, 18, 26]. We performed latero-lateral scanning in at least two interspaces of each field with longitudinal probe position and a representative image or clip was taken for offline validation. Posterior fields were examined only in the proximity of the posterior axillary lines, not requiring any important activity from the patient or the presence of an assistant to conform to the need of a reproducible situation during postoperative measurements even on mechanically ventilated patients.

The scans were performed three times on each patient. First, immediately before inducing anaesthesia in the operative theatre (preoperative). Second, within the first postoperative hour, but at least 15 min after the patients’ arrival to the postanaesthetic room or to the ICU to allow a phase for stabilization (postoperative 1 h). The third scan was performed 24 to 25 h after the second one (postoperative 24 h).

**Fig. 1:**
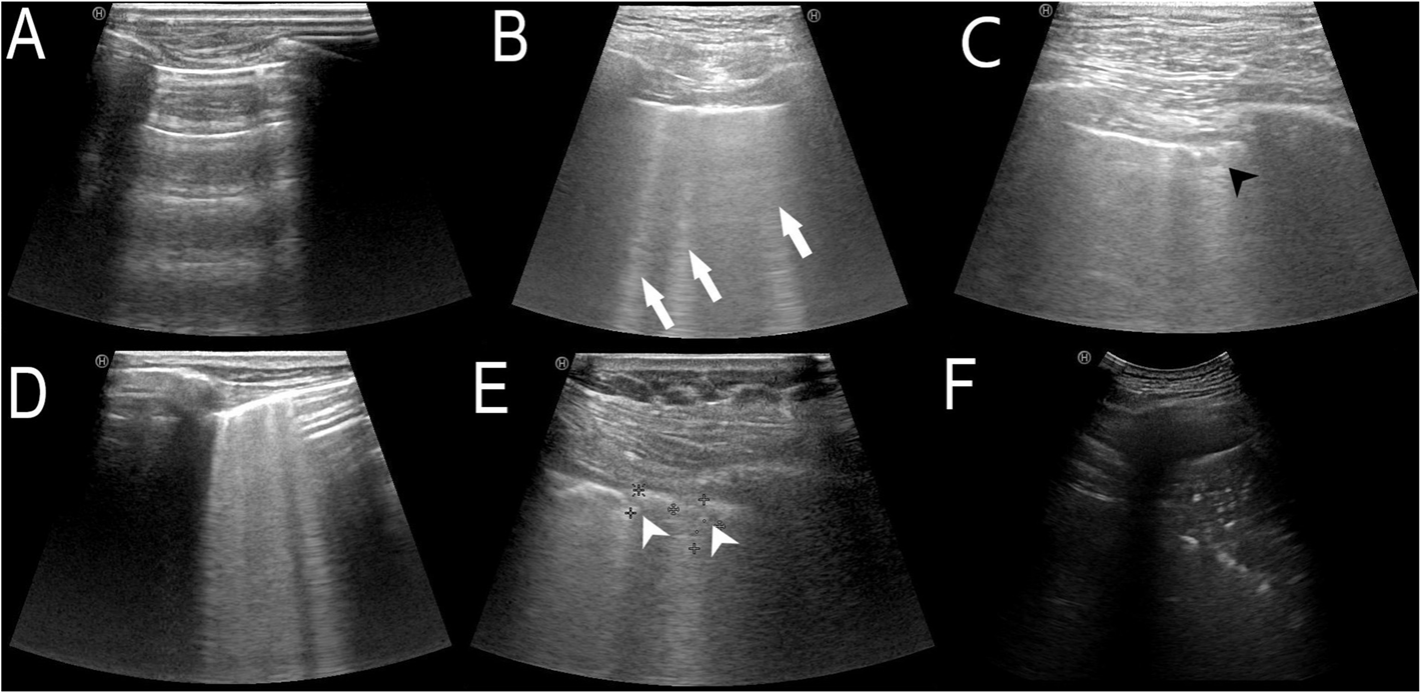
Typical ultrasound patterns with different scores in parentheses. **a**: A-profile (0 point); **b**: typical B-profile (1 point). B-lines marked with white arrows; **c**: small subpleural consolidation (black arrowhead) with clear pleural line (1 point); **d**: confluent B-profile (2 points); **e**: multiple subpleural consolidations (white arrowheads) and irregular pleural line (2 points); **f**: consolidated lung with aerobronchograms (3 points)

Lung ultrasound scores were calculated using a classification system optimized for perioperative settings described previously by Monastesse et al. [26]. A-profile was scored as 0 points, B-profile with more than 2 well-spaced lines/interspace or coalescent B-profile were registered as 1 or 2 points, respectively. For severe atelectasis with diameters exceeding 1 × 2 cm, 3 points were recorded. Small subpleural consolidations with clear pleural line were considered with 1, those multiple consolidations separated by an irregular pleural line with 2 points. The sum of these were calculated as lung ultrasound score (LUS) from 0 to 36. Typical ultrasonographic images for each profile are represented on Fig. 1. LUS calculation was done by the ultrasonographer, and a second observer validated it offline. In case of discrepancy, a third observer chose the final value from the available scores. At the defined postoperative time-points, absolute LUS and ΔLUS compared to the preoperative value were calculated. For picture archiving, we used a dedicated USB storage, LUS values were not provided to the attending staff members. When clinical conditions made a LU otherwise necessary (e.g. for ruling out pneumothorax) in the timeframe of a LU scheduled for study purposes, the focused data were provided and documented to patient records, while LUS values remained blinded.

### Anaesthesia protocol

Our institutional standards were followed. Preoperative epidural catheter insertion was performed in the operation theatre where appropriate. General anaesthesia was induced by administration of 1–2 μg/kg fentanyl and 1.5–2 mg/kg propofol at the discretion of the anaesthetist. Neuromuscular blockade for the endotracheal intubation was provided by either rocuronium or cis-atracurium selected upon the age and comorbid state. General anaesthesia was maintained with sevoflurane. For intraoperative ventilation, the tidal volume was set to 6–8 ml/kg based on ideal body weight, a respiratory rate was chosen to assure end-tidal CO_2_ of 35 ± 3 mmHg, the FiO_2_ was 0.4. PEEP was 5 cm of water. Episodes of intraoperative desaturation (SpO_2_ < 95% or > 3% decrease from initial) were managed as follows: the position of endotracheal tube was controlled by auscultation where appropriate, recruitment manoeuvre of manual inflation to at least 30 cm of water for 30 s was used, and PEEP was increased by 2 cmH_2_O, the additional increase of FiO_2_ was optional. Patients were awakened either in the operating room or in the intensive care. Residual neuromuscular blockade was reversed by 0.03 mg/kg neostigmine and 0.5 mg atropine if needed, based on clinical criteria or TOF values. Criteria for extubation on ICU followed our institutional routine involving normothermia (> 36 °C), adequate cooperation, and a favourable response to a spontaneous breathing trial of 30 min on PEEP of 5 cmH_2_O plus pressure support of a maximum of 10 cmH_2_O.

### Follow up for PPCs

The follow-up period for PPCs lasted 7 days postoperatively or until hospital discharge (the earlier completed). The check for PPCs was done by investigators unaware of LUS values and was based on patient records. No extra diagnostic or treatment activities were initiated by the investigators. The definitions included those of Canet et al. [2] including clinical and/or radiographic criteria: respiratory infection, respiratory failure, atelectasis, pleural effusion, bronchospasm, pneumothorax, and aspiration pneumonitis. Of note, screening was not limited to plain chest X-rays; all available medical imaging records were checked, and we added pulmonary oedema defined by presence of rales and tachypnoea with the need and suitable response to diuretics. The criteria of respiratory failure (PaO_2_ < 60 mmHg and/or SpO_2_ < 90% on room air and/or PaO_2_/FiO_2_ < 300 mmHg necessitating at least oxygen therapy) were extended by adding unplanned reintubation, need for non-invasive ventilation, or the inability to extubate a mechanically ventilated patient after 24 h. At the first verified PPC, we terminated the follow-up. Reoperation during the observation time resulted in exclusion, except in the cases where a case-definition of PPC was reached earlier. A PPC+ and a PPC- group were formed.

### Statistical analysis

#### Sample size

To calculate the sample size of the study, the absolute postoperative LUS was the variable of interest. We assumed that a minimum difference of 3 points was considered as clinically important, and that in combination with a standard deviation of 3 points were used for the calculations. This SD value resulted from a pilot study on 20 patients not involved in the study. A type one error of 0.05 and a required power of 0.80 were set. As unequal study groups were estimated with an approximate ratio of PPC+/ PPC- patients at 1 to 4, we used corrected sample sizes [30]. A minimum of 65 patients were required based on the conditions detailed above. To maintain adequate power in cases of loss for follow up or methodological failure, an additional 15% was screened, and a total of 76 patients were planned.

### Statistical analysis

Data were pooled for analysis in Microsoft Excel for Office 365, for the statistical analysis, we used StatsDirect 3.1.20 Statistical Software (Stats Direct Ltd., Grantchester, Cambridge, UK) following the same principles as in our previous works [31]. Continuous variables are presented as the means±standard deviation if they were normally distributed as tested by the Shapiro-Wilk W test. Non-normally distributed data are shown as the medians and interquartile ranges. Student’s two-sample t-test and the Mann-Whitney U test were used for comparisons as appropriate. Categorical variables are shown as percentages and absolute numbers of cases. The χ^2^ and Fisher exact test were used for contingency table analysis as appropriate. Variables with plausible impact on PPC risk (age, ASA class, BMI, congestive heart failure, COPD, diabetes, ongoing infection, smoking status, ARISCAT Score, SpO_2_, haemoglobin, creatinine levels, operative fluid balance, operation time, upper quadrant involvement, laparoscopy, LUS at 0 h, 1st and 24th postoperative hour) were all considered as candidates and were entered into a forward stepwise logistic regression model building approach to identify independent predictors in the study population (*p* for enter < 0.1, for exit > 0.1). Before model building, variance inflation factor (VIF) was calculated to estimate multicollinearity for each candidate continuous predictor. A predictor of VIF > 5 was considered as an indicator of serious collinearity and was excluded from further analysis. For these analyses, we used Dell Statistica 13.2 (Dell Inc., Tulsa, Oklahoma, USA). Odds ratios (ORs) and 95% confidence intervals (CI95%) were calculated. The Hosmer and Lemeshow statistic was used to assess model fit. For internal validation, a bootstrap method was used with 200 computer-generated samples. Two-sided *p*-values are shown, and the limit of statistical significance was set to *p* < 0.05. The diagnostic value of postoperative LUS was evaluated by calculating the sensitivity, specificity, and positive and negative predictive values at an optimal cut-off determined by the receiver operating characteristics (ROC) curve. The area under the plotted curve (AUC) was estimated by Wilcoxon’s method, and the standard error was calculated according to the method by DeLong. A bootstrap validation was performed for the confidence interval of the AUC as well.

## Results

**Fig. 2:**
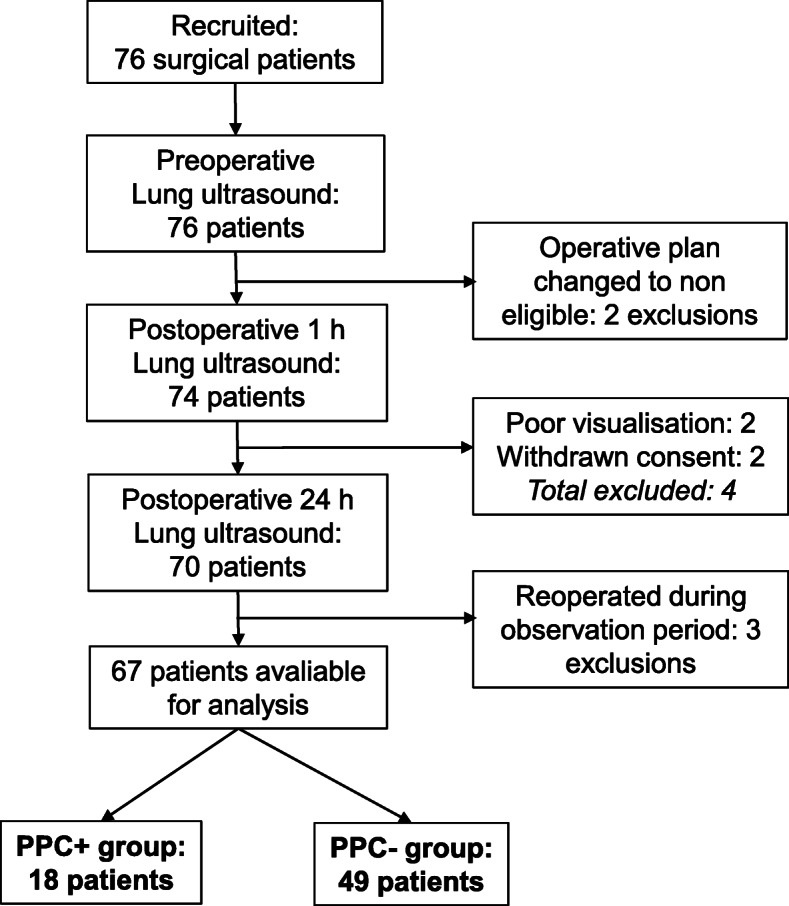
Study flowchart with reasons of exclusion in different stages

A total of 76 patients were enrolled. We had to exclude 9 previously eligible patients. Three of them were reoperated in the observation period, 2 withdrew consent, in 2 cases the follow-up scans were interfered by poor postoperative visualisation conditions, in 2 cases, the surgical plan was changed to a procedure not eligible for inclusion (in 1 case, previously unplanned thoracotomy was indicated because of surgical reason, in another case, an early near-fatal cardiac arrhythmia prevented further extension of the procedure). Finally, 67 subjects were available for analysis. Eighteen patients were assigned to the PPC+ group, 49 were evaluated in the PPC- population. Study flowchart is shown as Fig. 2.

The baseline characteristics of the PPC+ and PPC- groups are provided in Table 2. Most conditions were similarly represented in both groups, none of the potential predictors were significantly different. Patients conforming to ASA 3 class were significantly more represented in the PPC+ group.

**Table 2:** Baseline characteristics of the patients by group

Variable	PPC+ ***N = 18***	PPC- ***N = 49***	***p*** value
Age, years	68.4 ± 10.2	66.4 ± 9.6	0.4829
Male, N (%)	11 (61.1)	26 (53.1)	0.5570
ASA 3, N (%)	12 (66.7)	13 (26.5)	0.0026
BMI, kg/m^2^	26.4 ± 4.6	26.5 ± 5.5	0.9736
COPD, N (%)	5 (27.8)	5 (10.2)	0.1175
Hypertension, N (%)	11 (61.1)	33 (67.4)	0.6337
Congestive heart failure, N (%)	5 (27.8)	6 (12.2)	0.1494
Diabetes, N (%)	2 (11.1)	10 (20.4)	0.4903
Smoker, N (%)	2 (11.1)	5 (10.2)	1.0000
Active extrapulmonary infection, N (%)	3 (16.7)	6 (12.2)	0.6926
SpO_2_ on ambient air, %, median (IQR)	97 (91–94)	98 (92–96)	0.2588
Haemoglobin, g/dl	12.4 ± 2.5	13.0 ± 1.9	0.2892
Creatinine, μmol/l	86.2 ± 31.2	74.2 ± 18.3	0.1408

*ASA* American Society of Anaesthesiology, *BMI* Body Mass Index, *COPD* Chronic Pulmonary Disease, *IQR* interquartile range, *SpO*_*2*_ peripheral haemoglobin oxygen saturation

Operational data and characteristics available postoperatively are described in Table 3. Values of ARISCAT scores were significantly higher among PPC+ participants, otherwise, we did not detect important intergroup differences. Identified PPCs are provided in Table 4. The median time of onset of the PPCs was 2 days (IQR 1–3), 8 patients fulfilled PPC criteria on postoperative day 1.

**Table 3:** Postoperative characteristics of the patients by group

Variable	PPC+ ***N = 18***	PPC- ***N = 49***	***p*** value
Operation time, min, median (IQR)	190 (120–266)	123 (86–177)	0.0619
Surgeries with upper quadrant involvement, N (%)	14 (77.8)	35 (71.4)	0.7597
Upper gastrointestinal tract, N	4	9	
Pancreatic-biliary, N	7	16	
Liver resection, N	3	7	
Other, N	0	3	
Surgeries limited to lower quadrants, N (%)	4 (22.2)	14 (28.6)	
Colorectal, N	3	12	
Other, N	1	2	
Laparoscopy, N (%)	1 (5.56)	8 (16.3)	0.4258
Epidural catheter, N (%)	6 (33.3)	17 (34.7)	1.0000
Intravenous fluid, ml/kg/h, median (IQR)	10.7 (7.6–16.1)	10.9 (7.9–15.6)	0.9052
Estimated fluid balance, ml/kg, median (IQR)	22.4 (13.1–28.7)	19.1 (13–0-28.7)	0.1925
ARISCAT score	38 ± 12	25 ± 13	0.0006

*ARISCAT* Assess Respiratory Risk in Surgical Patients in Catalonia risk score, *IQR* interquartile range

**Table 4:** Type and frequency of detected PPCs

Type of PPC	N (%)
Respiratory failure	5 (27.8)
Pulmonary congestion	2 (11.1)
Pleural effusion (with or without atelectasis)	7 (38.9)
Bronchospasm	2 (11.1)
Respiratory tract infection	2 (11.1)

*PPC* Postoperative pulmonary complications

### LUS

**Fig. 3:**
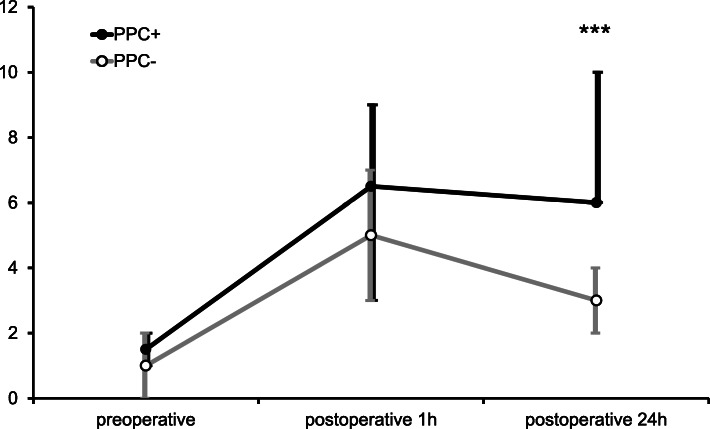
Lung ultrasound scores at different timepoints in the PPC+ and PPC- groups. Median values with interquartile ranges. ***: *p* < 0.0001 (Mann-Whitney U)

LUS kinetics are depicted on Fig. 3. No initial difference was present in terms of preoperative LUS values: the median LUS at 0 h was 1.5 (IQR 1–2) and 1 (IQR 0–2; *p* = 0.4625) in the PPC+ and PPC- groups, respectively. In the first postoperative hour, both groups had a marked increase, resulting in scores of 6.5 (IQR 3–9) and 5 (IQR 3–7). The value tended to be higher in the PPC+ group, but this difference was not significant (*p* = 0.1925). Median ΔLUS at this timepoint was + 5 (IQR 2–7) and + 3 (IQR 2–5), (*p* = 0.1765) respectively. When we compared the 24th postoperative hour’s LUS values, the persistently high scores in the PPC+ group (median 6; IQR 6–10) were significantly higher than those observed at PPC- participants (median 3; IQR 2–4; *p* < 0.0001). PPC+ patients had a median ΔLUS of + 5 as median (IQR 4–6) while PPC- subjects showed a close to complete remission with + 2 (IQR 1–3; *p* < 0.0001).

**Fig. 4:**
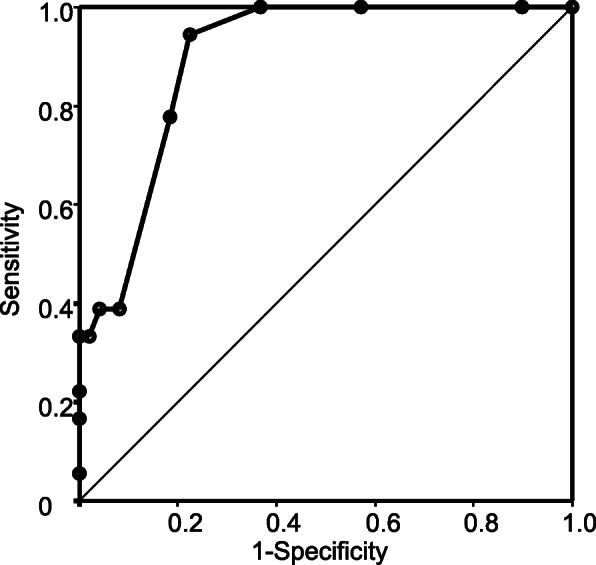
ROC curve of postoperative 24th hour lung ultrasound scores in the prediction of PPCs

Diagnostic performance of the 24th hour’s LUS are plotted for ROC on Fig. 4. The area under the curve was 0.8963 (CI95% 0.8253–0.9672). The bootstrap validation resulted a CI95% of 0.8158–0.9569. The optimal cut-off value was identified at LUS = 5. At this level, the sensitivity was 0.9444 (CI95% 0.7271–0.9986) with 0.7755 specificity (CI95% 0.6338–0.8823). Positive and negative predictive values were calculated as 0.6071 (CI95% 0.4058–0.7850) and 0.9744 (CI95% 0.8652–0.9994), respectively. This resulted in the likelihood ratio being equal to 4.2071 (CI95% 2.9704–5.9586).

### Multivariate analysis

Calculation of the variance inflation factors (VIF) showed that important collinearity was not found among continuous predictors: mean VIF was 1.8497, for individual VIFs, see Supplementary Table 1 in Additional file 2. There was no need for exclusion in this stage. In forward stepwise model building creatinine, LUS at 1 h and at 24 h were retained. Variables retained by the final model are reported in Table 5 with ORs. LUS at 1 h was not significantly associated with PPCs with an OR of 0.7232. By contrast, 24th postoperative hour’s LUS was verified to be an independent and significant risk factor for PPCs, having an OR of 2.6448. In our internal bootstrap validation the confidence intervals (CI95%) of the ORs were similar. Goodness of fit assessed by Hosmer Lemeshow had a *p* = 0.7804 suggesting good calibration.

**Table 5:** Odds Ratios of predictors for PPCs retained in the multivariate analysis

Variable	OR	CI95%	Bootstrap validated CI95%	***p*** value
Creatinine	1.0350	1.0022–1.0688	0.9957–1.0966	0.0364
LUS at 1 h	0.7232	0.4934–1.0599	0.4458–1.1629	0.0966
LUS at 24 h	2.6448	1.5555–4.4971	1.9341–4.2005	0.0003

*LUS* Lung Ultrasound Score, *PPCs* Postoperative Pulmonary Complications

## Discussion

The main objective of our study was to evaluate the value of lung ultrasonographic variables in a 24-h timeframe predicting PPCs. Our results confirmed that the concept of a quantitative lung-ultrasound-based scoring system is a valuable tool with high sensitivity and good specificity not only in the detection of full-born PPCs, but also for the identification of early phases of developing complications or patients at risk.

Lung ultrasound was proven to be valuable in screening for postoperative pulmonary pathologies after cardiac surgery with superiority compared to chest X-rays [15]. The quantitative evaluation of the lung deaeration is feasible in perioperative settings [26]. Therefore, the potential inclusion of this modality in a prediction model is an attractive option. Choosing a relatively rough end-point for their study, a French centre reported that patients postoperatively admitted to ICU needed more frequently postoperative ventilatory support, and had a lower PaO_2_/FiO_2_ ratio if their LUS was at least 10 immediately after admission [32]. In a recent study, the authors reported that among non-ICU postoperative patients, LUS can be a predictor of not only respiratory failure, but other PPCs as well. According to their results, higher postoperative LUS was typical in patients who developed PPCs [33]. PPC incidence in these reports (19–35%) was close to our observations (26.9%), slightly higher in an ICU population with ventilatory support. Of note, both study used the so-called original scoring system slightly different from ours, originated at Monastesse et al.’s modifications [26], as they did not score the small subpleural consolidations, which – together with the atelectatic areas in the proximity of the diaphragm – are proven to be important factors of perioperative loss of aeration [34]. At this point, we emphasize that our study is novel by means of introducing the protocol-based 24-h postoperative ultrasonographic follow-up. High LUS levels (12; IQR 7–18) suggested to be predictive for respiratory complications in previous reports were less frequent in our study, possibly due to the exclusion of thoracotomy patients and to the lower number of laparoscopic procedures [33]. In our study population, a transient increase in LUS at the earlier postoperative checkpoint did not increase risk of PPCs, but persistently elevated scores over 24 h identified a group of patients who are at significantly higher risk with high specificity and sensitivity. As more than a half of our patients in the PPC+ group did not complete any conventional PPC case definition at the time of the last LU, we underline the dual potential of our screening protocol in both the early detection and in the prediction of respiratory complications in the postoperative period.

The performance of LUS at 24 h after surgery as a prediction tool is worthwhile even in the light of previous models of the assessment of risk of PPCs. The area under the ROC curve in our study was 0.896 reflecting a strong prediction ability. This characteristic is similar to those reported in available risk stratification models validated in previous studies observing large populations [7, 10]. In an earlier paper, *McAlister* et al. reported an AUC of 0.875 for their model, which identified age, duration of anaesthesia, positive cough test, and nasogastric tubes to be independent predictors of PPCs [10]. Further excellent risk stratification models are currently available. For instance, the ARISCAT score uses seven easily accessible factors, and it was able to perform an area under the ROC curve of 0.90 [2]. This model was similarly effective in detecting high risk patients for respiratory failure [35] allowing anaesthesiologists to plan postoperative HDU/ICU admissions. However, this risk score was significantly higher in our PPC+ group, but did not qualify as an independent risk factor in our multivariate analysis, probably due to our inclusion criteria (especially the recruitment of patients undergoing procedures expected to be longer than 120 min), resulting both of our patient groups to have intermediate (> 25 points by definition) or high scores. Our LUS-based protocol can add further data and a decision point at 24 h after surgery for specific interventions, physiotherapy, and/or prolonged high dependency care. Of note, in our multivariate model, apart from LUS, preoperative creatinine level was also a mild risk factor, a finding hard to interpret in our study not focusing to the topic, while both PPC+ and PPC- groups had means in the normal range. Possible limited ability to empty extra fluid postoperatively can contribute to putting some patients at increased risk [36].

Our study has limitations. Our results cannot represent all our patients, as we had to limit our activity for definite study days. For a protocol potentially feasible to be used in everyday practice, we decided to avoid transducer changes, and a single linear probe was preferred; convex probes were only selected in case of poor visualisation especially at obese patients. This choice has also validation and allowed for our good imaging of the pleura [15, 24] but it could reduce our ability to detect some artefacts. For the same purpose, longitudinal scanning was chosen and ‘bat sign’ was our desired view. Even though more artefacts can be detected by transversal scanning with the whole footprint of a linear probe [18], this classical approach helped us not to create such a cumbersome protocol. Additionally, our scanning method examining 12 definite areas helped but did not completely ensure that repeated scans always insonate the same anatomical lung area, but this seemed to be feasible for frequent use. Excluding patients from analysis can always be perceived as a source of some bias, but repeated surgery could easily interfere with our protocol. LUS is prone to interobserver variability. For addressing this issue, we emphasize the importance of adequate training and the potential involvement of offline validation [28, 37]. Computer-aided measurement of B-lines and the percentage of the pleural line affected with these artefacts is reported to be a reproducible method with fast data analysis and showed a good correlation with the measured extravascular lung water or pulmonary capillary wedge pressure irrespective of ventilator settings [24, 38]. As our LUS method is optimized for perioperative use and relies equally on the assessment of even small consolidations, careful implementation of these algorithms is sought, but these promising tools are probably ahead of validation in this context and the automatization will be possible. The prevalence of poor postoperative visualization conditions completely preventing imaging was low in our population, but it may prevent the generalizability of our findings in profoundly different settings.

The optimal cut-off value from ROC analysis is probably specific to the study population, and also to the function of postoperative care. But the phenomenon of persistently high LUS at 24 h can focus attention on patients with increased risk of PPCs in a potentially reversible phase. Further studies should be initiated to identify optimal cut-offs for different postoperative populations.

## Conclusion

Persistently high postoperative lung aeration score at 24 h identify patients at risk of or in an early phase of postoperative pulmonary complications. Further investigation could implement these findings into the individualization of postoperative high-dependency care of these patients. We underline that LUS should be widely used and important efforts should be made for adequate training to have a valid, reproducible method in everyday use.

## Supplementary Information

**Additional file 1.** Dataset of the study. Categorical questions were marked with 1 (yes) or 0 (no). Abbreviations used in the table headers are explained as comments.

**Additional file 2.** Supplementary table.

## Data Availability

All data generated or analyzed during this study are included in this published article [and its Supplementary information files].

## Abbreviations

ARISCAT score: Assess Respiratory Risk in Surgical Patients in Catalonia score
ASA: American Society of Anesthesiologists
BMI: Body Mass Index
COPD: Chronic Pulmonary Disease
EF: Ejection Fraction
HDU: High Dependency Unit
ICU: Intensive Care Unit
IQR: Interquartile range
LU: Lung Ultrasound
LUS: Lung Ultrasound Score
PPC: Postoperative Pulmonary Complications
