# Supplementary material for: The role of ultrasonographic lung aeration score in the prediction of postoperative pulmonary complications: an observational study
Source: BMC Anesthesiol. 2021 Jan 14;21:19. doi: 10.1186/s12871-021-01236-6 (PMC7807225; doi:10.1186/s12871-021-01236-6)
Supplement: Supplementary file 2 — Additional file 2. Supplementary table. [file 12871_2021_1236_MOESM2_ESM.docx]

**Supplementary Table 1. Variance inflating factors**

| **Variable** | **Variance Inflating Factor (VIF)** |
| --- | --- |
| operation time | 3.0186 |
| ARISCAT Score | 2.7659 |
| estimated fluid balance | 1.9172 |
| LUS, preoperative | 1.8944 |
| Haemoglobin | 1.7057 |
| LUS at 1 h | 1.6792 |
| LUS at 24 h | 1.6283 |
| BMI | 1.6058 |
| creatinine | 1.4548 |
| age | 1.3855 |
| SpO_2_ | 1.2909 |
